# Development of an integrated milestone assessment tool across multiple early-adopter programs for breaking bad news: a pilot project

**DOI:** 10.1186/s12909-023-04715-1

**Published:** 2024-03-20

**Authors:** Anisha Turner, Sricharan Gopakumar, Charles Minard, Danielle Guffey, Nathan Allen, Dick Kuo, Kelly Poszywak, M. Tyson Pillow

**Affiliations:** 1 Baylor College of Medicine, One Baylor Plaza, Houston, TX 77030 USA; 2 Institute for Clinical and Translational Research, Baylor College of Medicine, Houston, TX USA; 3 Department Chair of Emergency Medicine at Baylor College of Medicine, Houston, TX USA; 4 Simulation and Standardized Patient Program at Baylor College of Medicine, Houston, TX USA

**Keywords:** Education environment, Evaluation, Integrated milestone assessment, Breaking bad news, SPIKES, Milestones 2.0, Interpersonal communication skills, Professionalism

## Abstract

**Background:**

The transition of the Accreditation Council for Graduate Medical Education (ACGME) to milestone assessment creates opportunities for collaboration and shared assessments across graduate medical programs. Breaking bad news is an essential communication skill that is a common milestone across almost every medical specialty. The purpose of this study was to develop and pilot an integrated milestone assessment (IMA) tool for breaking bad news using ACGME milestone criteria and to compare the IMA tool with the existing SPIKES protocol.

**Methods:**

The IMA tool was created using sub-anchors in professionalism and interpersonal communication skills that are applicable to every specialty and to the ability to break bad news. Two cases of breaking bad news, designed to be “easy” and “intermediate” in difficulty, were used to assess basic skills in breaking bad news in first-year medical residents from six residency specialties. Eight standardized patients were trained to portray the cases in sessions held in November 2013 and May 2014. Standardized patients completed an assessment checklist to evaluate each resident’s performance in breaking bad news based on their use of the SPIKES protocol and IMA tool. Residents answered post-encounter questions about their training and comfort in breaking bad news. The association between SPIKES and IMA scores was investigated by simple linear regression models and Spearman rank correlations.

**Results:**

There were 136 eligible medical residents: 108 (79.4%) participated in the first session and 97 (71.3%) participated in the second session, with 96 (70.6%) residents participating in both sessions. Overall, we were able to identify residents that performed at both extremes of the assessment criteria using the integrated milestone assessment (IMA) and the SPIKES protocol. Interestingly, residents rated themselves below “comfortable” on average.

**Conclusion:**

We developed an integrated milestone assessment (IMA) that was better than the SPIKES protocol at assessing the skill of breaking bad news. This collaborative assessment tool can be used as supplement tool in the era of milestone transformation. We aim assess our tool in other specialties and institutions, as well as assess other shared milestones across specialties.

## Practice points

1. The assessment of the ACGME core competencies interpersonal communication skills and professionalism is challenging in the clinical setting. The frequently required skills used when breaking bad news can be evaluated as a proxy to both competencies in nearly all medical specialties [1].
2. Milestones 2.0 emphasizes accurate evaluations, ideally through direct observational tools. Currently, no consistent process exists for summative and formative assessment of trainees regarding the skill of breaking bad news [2–4].
3. The Integrated Milestone Assessment (IMA) tool that we developed for breaking bad news would be a valuable addition to the Milestones 2.0 toolbox [3].
4. We developed an IMA tool that closely aligned with the well-accepted SPIKES protocol, yet the internal consistency and performance differentiation of our IMA tool surpassed that of the SPIKES protocol.

## Introduction

In 2013, the Accreditation Council for Graduate Medical Education (ACGME) transitioned to milestones as a competency-based assessment tool for medical resident trainees. While the competencies were crafted to provide a shared model of professional advancement among physicians in training, users found it difficult to understand the meaning of competencies in the context of their specialty [3, 5–9]. The resulting development of specialty-specific Milestones 1.0 lead to substantial variability in content and progression across milestone levels [7, 8]. These limitations led to the implementation of Milestones 2.0, which allows the use of more consistent, harmonized milestones and sub-competencies as quality assessment tools for programs to use across medical residency programs [7]. While there are several Milestone 2.0 assessment tools for the six ACGME core competencies, assessment of interpersonal communication skills (ICS) and professionalism (Prof) are particularly challenging in the clinical setting because of variations in faculty frames of reference and the influence of external-to-resident performance [7–9]. Faculty may use themselves, other doctors, or patient outcomes as frames of reference when assessing residents or may use their gut feeling or gestalt to translate their observations to numerical assessment scores [4].

Breaking bad news is an essential communication skill that is either explicitly included or strongly implied to be included in the ICS and Prof competencies and related milestones of nearly all medical specialties. While previous studies have utilized various tools to assess residents on the use of appropriate breaking bad news techniques, the development of a validated and simplified tool that includes direct observational assessment in any clinical setting and evaluates progress toward effective delivery of bad news would be valuable for all residency programs and would be a useful addition to the Milestones 2.0 toolbox [9–14]. Our objectives were to develop and pilot an integrated milestone assessment (IMA) tool for breaking bad news using ACGME milestone criteria, to compare this tool against the SPIKES protocol (the most commonly used and reported protocol for breaking bad news), and to assess medical residents’ self-perceptions of and comfort with their ability to break bad news [1, 10, 11].

## Background

Bad news is defined as any news that drastically and negatively alters the patient’s view of his or her future [15–17]. Breaking bad news in a compassionate way is an essential component of the doctor- patient relationship [18–20]. Nonetheless, most undergraduate and graduate medical programs lack formative or summative assessments for breaking bad news, leaving many physicians unprepared to handle such conversations with patients [18, 19]. Even experienced clinicians report that having to break bad news is a source of significant stress [4, 17–20]. Additionally, trainees enter residency programs with different levels of experience, as formal instruction during medical school for breaking bad news is highly variable [20]. Because most trainees lack previous hands-on experience, an intern’s first clinical experience of delivering bad news typically occurs during residency [17, 19]. This lack of practice and experience is less than ideal for patients and their families.

Despite the lack of training in medical school, residents are expected to use an appropriate technique for breaking bad news. Breaking bad news is a skill assessed in various ways for residency milestones, regardless of specialty, and is one of the top-three main themes in the assessment of ICS [5, 9, 15, 16]. Yet, the approach used to evaluate competency in breaking bad news is variable, subjective, and normative referenced.

To make assessments specific, objective, and criterion referenced, Milestones 2.0 encourages the development of validated assessment tools that would inform an institution’s clinical competency committee of the proficiency level within each sub-competency [3]. The SPIKES protocol is a widely recognized six-step protocol that was first developed in 2000 to deliver bad news to cancer patients. It has been adopted more widely and is now used by clinicians in various settings to communicate bad news to patients [1] in a clear, compassionate, and supportive manner. As the most popular protocol for delivery of difficult news, the SPIKES protocol has reached guideline status in the United States and a number of other countries and is used as a training guide for communication skills [1]. The SPIKES acronym stands for the following steps: Setting, Perception, Invitation, Knowledge, Empathy, and Summary. Although there are several other protocols for the delivery of bad news, such as GRIEV_ING, the ABCDE approach, and the BREAKS protocol, a review of the literature shows SPIKES to be one of the preferred protocols for teaching students to deliver bad news [10, 11, 21]. To our knowledge, a shared common assessment tool incorporated into the sub-competencies of the Milestone 2.0 ICS and Prof competencies does not exist. This project sought to pilot an innovative, harmonized ACGME milestone-based IMA tool across six of seven early-adopter programs at a single institution to evaluate medical resident proficiency in communication of bad news and to compare this new tool to the established SPIKES protocol.

## Methods

This study was conducted at a single institution and qualified for institutional review board exemption. No formal training in breaking bad news existed for residents at the time of study. The milestones from six of seven early-adopter programs (emergency medicine, medicine, neurosurgery, orthopedics, radiology, and urology) were reviewed to find the most common assessment themes across programs. The breaking bad news sub-competency was present across the six programs, but at varying levels in each of the milestones and sub-competencies. Thus, instead of using each specialty's milestones for their respective residents, sub-anchors in Prof and ICS applicable to every specialty were created and labeled as IMAs (Table 1). Additionally, the SPIKES protocol was used as a measurement tool to lend validity evidence to our milestone assessment (Table 2).

**Table 1:**
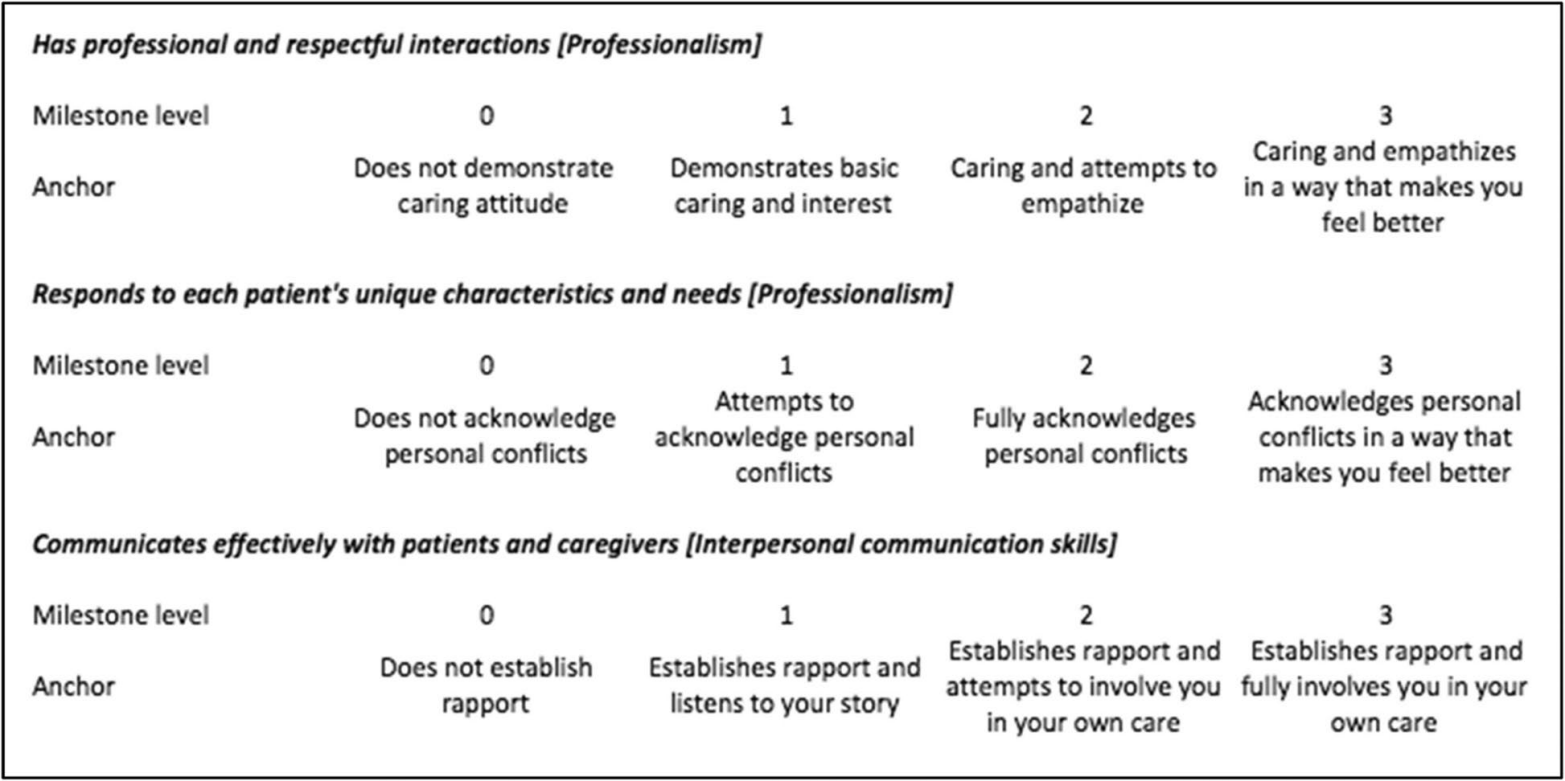
Integrated milestone assessment anchors (Supplemental content)

**Table 2:**
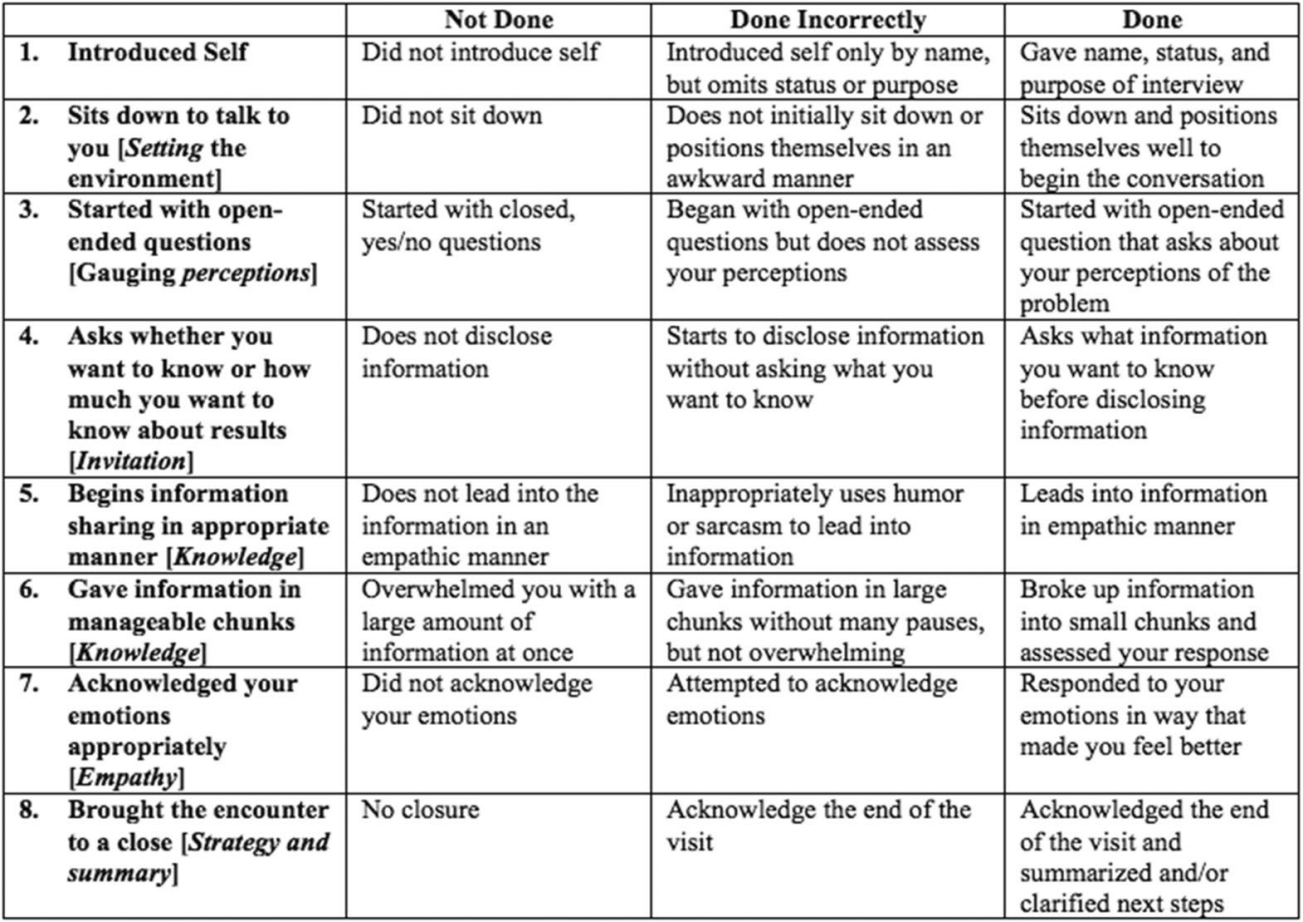
SPIKES protocol (Supplemental content)

Two cases of breaking bad news were designed as part of the pilot formative assessment project to evaluate medical residents’ skills in breaking bad news. The two cases varied in difficulty, with one basic case designed to be of “easy” difficulty and another more challenging case of “intermediate” difficulty. Two separate sessions with standardized patients (SPs) were held to assess the skills of residents across the six specialties, which each learner taking the “easy” case followed by the “intermediate” case. The sessions were held in November 2013 (5 months into training) and in May 2014 (10 months into training), based largely on convenience of scheduling. Eight professional and experienced SPs were trained before each session by the medical director of the simulation center to ensure consistency in the cases and resident feedback for each session. The SPs completed two rubric-based assessments centered on the milestones’ competencies and on the SPIKES protocol for each resident. Finally, residents received feedback on their performance and answered post-encounter questions about their training and comfort in breaking bad news after each case.

Statistical analysis was performed using SAS software. The association between SPIKES and IMA scores was investigated by simple linear regression models, stratified by session. Spearman rank correlations were also estimated to describe the strength of the linear association between scores. Cronbach’s alpha was used to measure the internal consistency of the SPs responses on each scoring mechanism, with the alpha values being 0.61 (session one) and 0.44 (session two) for the SPIKES protocol and 0.91 (session one) and 0.83 (session two) for the IMA tool. Item deletion was used to investigate the relative item contribution to the overall scores. A general linear mixed model was used to test for significant changes in SPIKES and IMA scores between sessions. The model included fixed effects for session (discrete), and the residuals assumed an unstructured matrix of correlated error terms. Separate models were fitted for each score. A Bland–Altman analysis with 95% limits of agreement was used to investigate agreement between the SPIKES and IMA scores, stratified by session, by comparing differences in standardized scores. The scores were standardized within each session by subtracting the mean score and then dividing by the standard deviation of the mean score (SD).

## Results

There were 136 eligible residents across the six early-adopter programs: 108 (79.4%) residents participated in the first session and 97 (71.3%) residents participated in the second session, with 96 (70.6%) residents participating in both sessions. Table 3 summarizes the demographics of the participants. Table 4 summarizes the SPIKES score and IMA score for each of the two sessions.

**Table 3:** Overall demographics of session participants

	**n**	**%**
Number of first-year residents in early-adopter programs	136	-
Participants in pilot	108	79.4%
Female participants	46	42.6%
**Specialties**		
Emergency medicine	13	12.0%
Internal medicine	67	62.0%
Neurosurgery	3	2.8%
Orthopedic surgery	5	4.6%
Radiology	12	11.1%
Urology	4	3.7%

**Table 4:** Summary of performance based on SPIKES vs. IMA assessment tools

	Session one *n* = 108		Session two *n* = 97		*P*-value
	Average	SD	Average	SD	
SPIKES protocol					
Introduced self	0.91	0.19	0.94	0.17	-
Setting the environment	0.97	0.13	0.99	0.05	-
Gauging perceptions	0.92	0.22	0.91	0.22	-
Invitation	0.16	0.37	0.10	0.31	-
Knowledge 1	0.92	0.27	0.96	0.18	-
Knowledge 2	0.86	0.25	0.84	0.27	-
Empathy	0.84	0.25	0.77	0.25	-
Strategy and summary	0.99	0.28	0.99	0.05	-
Mean total score (out of 8)	6.56	0.98	6.52	0.75	0.70
Integrated Milestone Assessment					
Professional and respectful interactions (Prof)	2.3	0.8	2.2	0.6	-
Responds to each patient’s needs/characteristics (ICS)	2.2	0.9	1.8	0.8	-
Communicates effectively with patients/caregivers (ICS)	2.3	0.8	1.9	0.8	-
Mean total score (out of 9)	6.81	2.35	5.82	1.93	< 0.001

For the SPIKES protocol, the overall Cronbach’s alpha values were 0.61 (session one) and 0.44 (session two). For the IMA tool, the overall Cronbach’s alpha values were 0.91 (session one) and 0.83 (session two).

In session one, 4 residents (3.8%) scored 0 in at least one of the three milestone areas and 44 residents (42.3%) scored 3 in all three areas. On a 1–5 Likert scale with 5 being “comfortable,” residents rated their ability to break bad news as a mean of 3.5 (SD 0.8) and their ability to deal with patients’ emotions as a mean of 3.7 (SD 0.8). In session two, 4 residents (4.1%) scored 0 in at least one of the three milestone areas and 9 (9.3%) scored 3 in all three areas. Just over half (55.7%) of the residents reported that they had not received any formal training in breaking bad news during their residency, although the large majority reported either informal training or modeling from other residents or attending physicians.

**Fig. 1:**
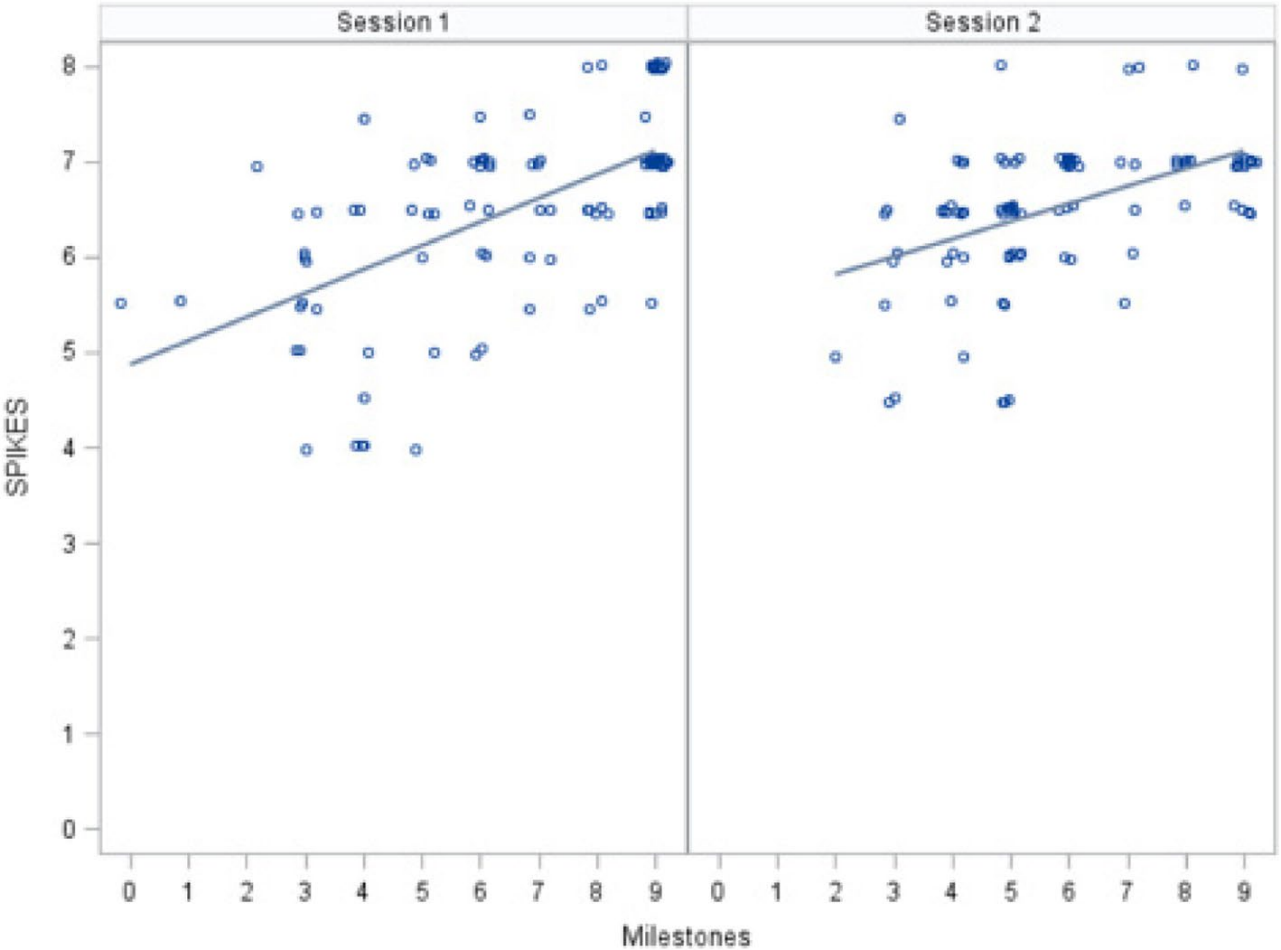
Linear regression of SPIKES to Integrated Milestone Assessment (IMA) Scores (supplemental content)

There was no statistically significant difference in SPIKES scores between sessions (*P* = 0.70). The mean SPIKES scores were 6.56 (SD 0.98) for session one and 6.52 (SD 0.75) for session two. However, there was a statistically significant difference in IMA scores between sessions (*P* < 0.001). The mean IMA scores were 6.81 (SD 2.35) for session one and 5.82 (SD 1.93) for session two. The mean difference was 1.02 points (95% CI: 0.48, 1.55). Figure 1 compares SPIKES and IMA scores for each resident stratified by session using a simple linear regression. IMA scores were significantly associated with SPIKES scores for session one (slope = 0.25, *P* < 0.001) and session two (slope = 0.19, *P* < 0.001). The correlation between scores was 0.62 and 0.53 for sessions one and two, respectively. Lastly, there was greater variability (SD) for the IMA tool total scores compared to the SPIKES protocol total scores for both sessions (Table 4).

## Discussion

The scores generated from the SPIKES protocol and the IMA tool were moderately and positively correlated. Because the SPIKES protocol is a recognized step-by-step checklist for breaking bad news, a positive correlation supports the use of the IMA tool to assess the skill of breaking bad news. Furthermore, we believe the IMA tool is better than the SPIKES protocol, because the IMA scores more accurately reflected the difficulty of the case and because the IMA scores were internally consistent, as measured by Cronbach’s alpha. Specifically, there was a statistically significant difference in IMA scores, but not SPIKES scores, between the two sessions, suggesting that the IMA tool better differentiates between high and low performance.

The Cronbach’s alpha values for the SPIKES scores suggest relatively poor internal consistency (i.e., reliability), which could be caused by the relatively small number of items included in the checklist, poor similarity between items, or heterogeneous constructs (i.e., there may be more than one latent variable [multidimensionality] described by the SPIKES score). Item deletion analysis did not identify a component of the SPIKES score that was especially important or that could be removed from session one. However, removing “invitation” (SPIKES protocol #4, Box 2) from session two scores would increase Cronbach’s alpha to 0.57 (from 0.44). In contrast, the Cronbach’s alpha value from the IMA scores suggests a strong internal consistency (i.e., reliability) and surpassed that of the broadly used SPIKES protocol. Again, item deletion analysis did not identify a component of the IMA score that was especially important or that could be removed from either session.

In the context of Milestones 2.0, the goal is to develop shared assessment tools that can be used across specialties to accurately evaluate skills. It is our belief that IMA can be used and applied to all specialties despite the appearance of the skill of breaking bad news in multiple milestone levels across the specialties [2, 7]. As Milestones 2.0 develops more harmonized sub-competencies and sub-anchors, we propose that the IMA tool could be used to further evaluate breaking bad news as a skill commonly listed in the ICS and Prof competencies. Additionally, the IMA tool could be an excellent evaluation tool for medical students and residents to assess their skills of breaking bad news in objective structured clinical examinations or in bedside evaluations, as SP encounters with validated tools is an established method of assessing learners [7, 22]. The data gathered could be useful to quantitate performance to an institution’s clinical competency committee and could be used to provide consistent and reliable quantitative data to medical residency program directors. We believe that the incorporation of a format similar to Milestones 2.0 makes the IMA tool more familiar and user-friendly for graduate medical education evaluators compared with the format of the SPIKES protocol.

### Limitations

Our study is limited in its design methodology, as it was a pilot study and tested two different difficulty levels of cases at two sessions during the medical residency year. As this study was a pilot study, all participants remained anonymous at the request of the Office of Graduate Medical Education, Baylor College of Medicine, Houston, TX, USA. This anonymity prevented us from performing a matched-pair analysis on the trainees’ data. The way the cases were tested also limited our design quality, as either the "easy" case and the "intermediate" case should have been tested at the same time or one difficulty level should have been tested at separate times. Also, residents break bad news routinely, despite the lack of formal teaching in breaking bad news; thus, some residents may gain more practice and proficiency in breaking bad news over time than others. Furthermore, residents may have received training in medical school or prior exposure to clinical settings. Additionally, the data from both sessions was reviewed retrospectively. Another limitation is that, since the sessions were months apart, the SPs knew there had been a previous encounter which could have injected bias into the second session. Finally, we asked post-encounter questions regarding residency background and comfort with breaking bad news but did not ask pre-encounter questions.

## Conclusions

While the skill of breaking bad news is variably present across competencies in Milestone 1.0, this skill is one that is shared by all specialties and is an excellent target for Milestone 2.0’s goal of harmonized assessments. Currently, there is no standardized approach to assess the skill of breaking bad news. We developed the IMA tool to evaluate the delivery of bad news, and it would be a valuable addition to the Milestones 2.0 toolbox. A simplified method, such as the IMA tool, may be easier to use than the SPIKES protocol and may also address more milestone language, making it more valuable to clinical competency committees and more in agreement with Milestone 2.0 requirements. Finally, breaking bad news, while explicitly mentioned in the majority of specialties’ milestones, is not the only skill in the ICS and Prof competencies. By broadening the assessment and language, it might be possible to develop a tool that can assess specific skills required to meet the competencies and the broader skills required to meet the milestones. Our data are valuable to medical residency programs across the United States to further develop and test a shared assessment for breaking bad news that can also be used in milestone assessments by clinical competency committees. As such, the IMA tool demonstrates promise as an effective evaluator of medical resident proficiency in breaking bad news. Additionally, the concept and theoretical underpinnings of this study are essential for the advancement of learner assessment and can serve as a catalyst for further advancements in graduate medical education.

## Data Availability

The authors confirm that the data supporting the findings of this study are available within the article.
